# A Complete Telomere‐To‐Telomere Assembly of *Plectropomus leopardus* and Phylogenomic Insights Into Perciformes

**DOI:** 10.1111/eva.70296

**Published:** 2026-07-09

**Authors:** Baojun Zhao, Chaofan Jin, Mingjian Liu, Haizhan Tang, Da Zheng, Mengya Wang, Bo Wang, Zhenmin Bao, Jingjie Hu

**Affiliations:** ^1^ MOE Key Laboratory of Marine Genetics and Breeding, College of Marine Life Sciences/Key Laboratory of Tropical Aquatic Germplasm of Hainan Province, Sanya Oceanographic Institution, Ocean University of China Qingdao China; ^2^ Southern Marine Science and Engineer Guangdong Laboratory Guangzhou China

**Keywords:** Epinephelidae, Perciformes, phylogeny, *Plectropomus leopardus*, T2T genome

## Abstract

The leopard coral grouper, *Plectropomus leopardus* Lacepède, 1802, is an ecologically and economically important species in the family Epinephelidae. However, the absence of a complete reference genome has impeded advanced genetic research on this species, and the phylogenetic position of Epinephelidae within Perciformes remains unresolved. Here, we generated a complete telomere‐to‐telomere (T2T) genome assembly (887.20 Mb; contig N50: 39.03 Mb; QV: 67) for *P. leopardus* with no need for gap‐filling. Leveraging this genome combined with other available genome resources, we conducted a large‐scale genomic re‐annotation and reconstructed a robust genome‐level phylogeny of the Perciformes. Our analysis revealed that Epinephelidae and Anthiadidae form a monophyletic clade situated at the base of the Perciformes, indicating that the traditional suborder Serranoidei is non‐monophyletic. Comparative genomic analysis revealed that differential expansions of transposable elements represented the primary driver of genome size variation among *P. leopardus* and other groupers. Furthermore, we constructed a high‐density haplotype reference panel using 110 individuals and annotated 1068 loss‐of‐function (LoF) mutations. Enrichment analysis indicated that genes with stop‐gain LoF mutations are involved in the immune response and skin color variation. This study provides an essential genomic resource for *P. leopardus* and offers fundamental evolutionary insights into the Epinephelidae and, more broadly, the order Perciformes.

## 1 Introduction

The leopard coral grouper *Plectropomus leopardus* Lacepède, 1802, also known as the common coral trout, is a representative species of the genus *Plectropomus* (family Epinephelidae, order Perciformes). The species naturally inhabits tropical or subtropical waters of the Indo‐Pacific region, ranging from southern Japan to Australia, and eastward to the Caroline Islands and Fiji (Yang et al. 2020). Within coral reef ecosystems, *P. leopardus* functions as an apex predator, playing a crucial role in maintaining ecological balance. While juveniles (total length < 12 cm) primarily consume a high proportion (44%) of benthic crustaceans, the proportion of fish consumption increases substantially as they grow, resulting in adult individuals that are almost entirely (95%) piscivorous (Frisch et al. 2016).

Like most groupers (Epinephelidae), *P. leopardus* is an economically significant species (Astari et al. 2025). This species is highly popular in the market due to low‐fat and high‐protein flesh, taste, and bright red coloration, with prices potentially reaching $50–75/kg (Burgess et al. 2019). The high value and market demand have resulted in heavy exploitation by commercial fishing ventures across the Pacific and Indian Oceans. Overfishing has depleted populations of *P. leopardus* throughout Southeast Asia, prompting the relocation of fishing efforts to Australian waters (Carter et al. 2014). To address this demand, commercial aquaculture and breeding programs are rapidly emerging (Astari et al. 2025; Liu et al. 2025; Wang, Xin, et al. 2024). Concurrently, extensive genetic research on this species is actively being conducted; for instance, identification of candidate genes for growth (Wang et al. 2023), skin color (Yang et al. 2020), and disease resistance (Wang, Yu, et al. 2024). Although several draft genomes of *P. leopardus* have been reported (Han et al. 2023; Zhou et al. 2020), the assemblies remain incomplete, which could introduce bias into trait dissection and genetic breeding studies. Providing a complete T2T genome remains essential and urgent for advancing molecular breeding programs, as well as for the conservation of this species.

From a broader taxonomic perspective, the order Perciformes, to which the leopard coral grouper belongs, has long been treated as a taxonomic “wastebasket” in fish systematics (Betancur‐R et al. 2017). Any lineage of percomorph that was not as morphologically distinctive as a flatfish or seahorse was classified into this order. By the end of the 20th century, Perciformes encompassed over 10,000 species distributed across 160 families (Ghezelayagh et al. 2022). Molecular phylogenetic studies have since prompted a comprehensive redefinition of the group (Betancur‐R et al. 2017). The newly defined Perciformes now comprises more than 3200 species classified into at least 53 taxonomic families. Although the phylogeny of Perciformes has been investigated using certain molecular markers, the phylogenetic relationships among its suborders and families remain uncertain, particularly regarding Serranoidei and its constituent taxa (Betancur‐R et al. 2017; Ghezelayagh et al. 2022; Hughes et al. 2018). With the rapid advancement of sequencing technologies, genome data for species within Perciformes have surged exponentially, providing an unprecedented opportunity to gain phylogenomic insights into this group (Acero et al. 2024; Fuhrmann et al. 2024; Holland 2025; Zhao et al. 2025). However, the lack of genome annotations for certain species has hindered both data integration and evolutionary insights into this taxonomic group.

In this study, we generated a complete T2T genome assembly for the leopard coral grouper. By leveraging this reference genome combined with other available genome resources, we conducted a large‐scale genome re‐annotation and reconstructed a robust genome‐level phylogeny of Perciformes, which provides new insights into the evolutionary history and taxonomic relationships of this diverse fish order. Furthermore, we investigated the dynamics of transposable elements (TEs) activity, identifying differential transposon expansions as the primary driver of genome size variation among groupers. Finally, we constructed a high‐density haplotype reference panel and performed detailed annotation of loss‐of‐function mutations. Our results not only provide important genome resources for *P. leopardus* , but also offer new insights into the genome evolution of Epinephelidae, as well as of Perciformes.

## 2 Methods

### 2.1 Sample Preparation and Sequencing

A 3‐year‐old specimen of *P. leopardus* was collected from Lingshui Delin Aquaculture Co. Ltd. The individual was euthanized with 250‐mg/L tricaine methanesulfonate (MS‐222, Sigma, USA). The dissected tissue samples were rapidly frozen in liquid nitrogen and stored at −80°C for long‐term preservation. Total genomic DNA was prepared by the nuclei method for ONT ultra‐long sequencing. The quantity and integrity of the total DNA were evaluated with Qubit 3.0 and pulsed‐field gel electrophoresis, respectively. High‐quality DNA (main band > 100 Kb) was used for library construction with SQK‐LSK114 (V14) ligation sequencing kit. Sequencing was performed with the R10.4.1 flow cell. Basecalling was performed using Dorado v0.9.0 with the *dna_r10.4.1_e8.2_400bps_sup@v5.0.0* model (Supplementary File 1). Subsequently, ONT reads with lengths ≥ 30 kb and base quality ≥ Q20 were retained. Error correction of ONT sequencing data was performed using HERRO. For PacBio sequencing, a > 15‐kb SMRTbell library was constructed and sequenced on the PacBio Revio platform, and reads with low quality (Q20) and lengths < 10 kb were filtered out. A Hi‐C library was constructed using muscle tissue from the same specimen, following the standard MboI restriction enzyme protocol, and sequenced on the DNBSEQ‐T7 platform. The quality control of Hi‐C data was performed using fastp v0.24.0 with “–l 100.”

### 2.2 Genome Assembly

The Raft_hifiasm pipeline was employed for genome assembly (Cheng et al. 2022; Kamath et al. 2024). Briefly, HERRO‐corrected ultra‐long (UL) ONT reads were further corrected using Hifiasm v0.20.0‐r639 and subsequently fragmented into 20‐kb reads. These fragmented 20‐kb ONT reads were then combined with > 13‐kb PacBio HiFi reads and assembled using Hifiasm with the following parameters: –telo‐m CCCTAA, along with HERRO‐corrected ultra‐long reads (–ul) and Hi‐C data (–h1/–h2). To achieve greater chromosome completeness and continuity, two additional genome versions were generated, each yielding two haplotype‐resolved assemblies (six in total). Alternative Assembly v2 was constructed using ~20× coverage of 20‐kb fragmented ONT reads combined with > 15‐kb PacBio HiFi reads, and incorporated HERRO‐corrected ultra‐long reads, Hi‐C data, and modified Hifiasm parameters (–s 0.50 –D 6 –N 105 –telo‐m CCCTAA). Alternative Assembly v3 followed the standard Hifiasm method (HiFi+UL), utilizing > 10‐kb HiFi reads and > 50‐kb Q20 ONT reads (–ul) along with Hi‐C data and the parameters –s 0.50 –D 6 –N 105 –telo‐m CCCTAA (Cheng et al. 2022). All assemblies were scaffolded using 3D‐DNA v180114 with the parameter “–r 0.” Then Juicebox v1.11.08 was used to manually review and check assembly errors, retaining only scaffolds composed of a single contig (Durand et al. 2016). Subsequently, the contigs were subjected to telomere identification with quartT2T (Lin et al. 2023), with only those exhibiting complete telomeres at both ends retained. The final assembly was generated by extracting complete chromosomes from the six haplotype‐resolved assemblies, and retained only the best representative for each chromosome. This yielded a high‐quality genome comprising 24 gapless contigs, each corresponding to a complete chromosome. The genome quality value (QV) of the genome assemblies was assessed using Merqury v1.3 with PacBio HiFi reads. The assembly and annotation completeness were evaluated by Benchmarking Universal Single‐Copy Orthologs (BUSCO) v5.8.2 against actinopterygii_odb10 (2024‐01‐08). Structural variations between our genome and the previous version (Han et al. 2023) were detected using Syri v1.7.1 with default parameters, and the results were plotted using Plotsr v1.1.1 (Goel et al. 2019).

### 2.3 TE Annotation and Evolution

RepeatModeler v2.0.5 was employed to construct a de novo TE library for *P. leopardus* (Flynn et al. 2020). Additionally, LTR_FINDER v1.0.7 (parameters: –C –d 1000 –L 7000 –l 100 –p 20 –M 0.9) and LTR_retriever v3.0.1 were utilized to build an LTR retrotransposon library (Ou and Jiang 2018; Xu and Wang 2007). Subsequently, CD‐HIT was applied to remove redundant sequences from the combined two libraries. Finally, RepeatMasker v4.1.7 was used to perform soft masking of the genome with the parameter “–gff –xsmall.” To evaluate the evolutionary history of repetitive sequences in *P. leopardus* and its closely related species, *Cephalopholis sonnerati* Valenciennes, 1828, and *Epinephelus corallicola* Valenciennes, 1828, were also processed using the above pipeline.

The divergence of TEs from consensus sequences was adjusted for multiple substitutions using the Jukes–Cantor model: *K* = −(3/4) × ln(1 − D × 4/3). The insertion time of TEs was calculated using the equation *T* = *K*/2*r*, where *T* denotes the insertion time and *r* represents the nucleotide substitution rate. The mutation rate per generation was determined using three Percomorphaceae species, including *Amphiprion ocellaris* Cuvier, 1830 (7.94 × 10^−9^), *Cynoglossus semilaevis* Günther, 1873 (8.62 × 10^−9^), and *Paralichthys olivaceus* Temminck & Schlegel, 1846 (7.95 × 10^−9^) (Bergeron et al. 2023). The average per‐generation mutation rate was calculated as 8.17 × 10^−9^. Assuming an average generation time of 3 years for groupers (Ding et al. 2024; Phạm et al. 2022), the mutation rate r was estimated to be around 2.7 × 10^−9^ per site per year.

### 2.4 Coding‐Gene Prediction and Function Annotation

The repeat‐masked genome assembly was used for gene annotation with the BRAKER v3.0.8 pipeline (Gabriel et al. 2024), which integrates evidence from homology‐based prediction, transcriptome‐guided prediction and ab initio gene prediction (Brůna et al. 2024; Gabriel et al. 2021; Stanke et al. 2006). The transcriptome data were from NCBI BioProject PRJNA1051334 (Jin et al. 2024). The homologous protein datasets encompassed genome annotation files from three species: *Danio rerio* Hamilton, 1822 (GCF_000002035.6), *Epinephelus lanceolatus* Bloch, 1790 (GCF_005281545.1), and *Centropristis striata* Linnaeus, 1758 (GCF_030273125.1). The UTR annotation of the genome was performed using the PASA_pipeline with the transcripts from the results of Trinity v2.14 (Grabherr et al. 2011; Haas 2003). Additionally, the previous genome annotations file was subjected to coordinate transposition using Liftoff v1.6.3 (Han et al. 2023), and integrated using Bedtools “intersect –v” with the annotations from BRAKER3 + PASA. Distribution of TEs and gene density across the genome was visualized using Circos v0.69.

Functional annotation for the predicted protein‐coding genes was conducted employing EggNOG Mapper using the EggNOG database with mmseqs (Huerta‐Cepas et al. 2019). This analysis provided annotations across diverse categories, including gene names, Gene Ontologies (GOs), enzyme functions (EC), KEGG pathways, CAZy families, PFAM domains, and more.

### 2.5 Phylogenomic Analyses

To establish a robust phylogenomic framework for the order Perciformes, this study performed comprehensive re‐annotation of publicly available chromosome‐level genomes and high‐quality scaffold‐level genomes (N50 > 1 Mb and genome BUSCO > 90%) from NCBI that lacked genome annotations. For genomes without transcriptome support, annotation was carried out using transcriptome data from closely related species or through homology‐based prediction. To improve the completeness of annotation, BRAKER‐derived gene annotations were combined with “genemark_supported.gtf” from GeneMark (Brůna et al. 2024).

Single‐copy orthologs were identified using OrthoFinder v2.5.2 (Emms and Kelly 2015). Multiple sequence alignment for each ortholog was performed with MAFFT under the “–maxiterate 1000 –globalpair” parameter. Poorly aligned positions were subsequently trimmed using Gblocks v0.91b with parameters “–t = *p* –b5 = *n*.” Trimmed alignments were concatenated into a supermatrix using FASconCAT‐G v1.05. The best fitted model was selected under the Akaike Information Criterion (AIC) via ModelTest‐NG v0.1.7, utilizing the –T raxml/mrbayes implementation (Darriba et al. 2020). Maximum likelihood phylogeny reconstruction was executed in RAxML‐NG v1.1.0, employing 1000 bootstrap replicates for branch support estimation (Kozlov et al. 2019). Bayesian phylogenetic inference was carried out using MrBayes v3.2.7 (Altekar et al. 2004). Two independent Markov chain Monte Carlo simulations were performed with four (one cold and three hot) chains for 5,000,000 generations, sampling every 500 generations. The initial 25% of sampled trees were discarded as burn‐in.

The divergence time estimation was inferred using MCMCtree of PAML v4.9J (Yang 1997). The phylogenetic tree was calibrated using 6 nodes: (1) The root node divergence between Carangaria (outgroups) and Eupercaria was constrained to have occurred 95.4–109.0 Mya, with a 95% confidence interval, based on molecular clock analyses from a previous study (Matschiner et al. 2020). (2) The crown group of Perciformes was constrained with a minimum age of 61.6 Mya and a 95% soft upper boundary at 72.1 Mya according to the fossil records (Schwarzhans et al. 2024; Schwarzhans and Jagt 2021) and molecular clock analyses (Ghezelayagh et al. 2022). (3) The divergence between Tetraodontiformes and Lophiiformes was constrained with a minimum hard age of 55.8 Mya, based on †*Balkaria histiopterygia* (https://fishtreeoflife.org/fossils/). (4) The stem group of Scorpaenoidei was calibrated with a hard minimum age of 48.07 Mya, based on three fossils from the Ypresian Stage (https://paleobiodb.org/). (5) The Most Recent Common Ancestor (MRCA) of the clade comprising Pleuronectidae and Paralichthyidae was constrained with a minimum age of 29.62 Mya and a 95% soft upper boundary at 35.3 Mya, based on †*Oligopleuronectes germanicus* from the Frauenweiler fossil site, Germany (Ghezelayagh et al. 2022; Harrington et al. 2016). (6) The MRCA of *Gasterosteus aculeatus* Linnaeus, 1758 and *Pungitius pungitius* Linnaeus, 1758 was constrained with a minimum age of 13.1 Mya and a 95% soft upper boundary at 39.39 Mya, based on the †*Gasterosteus cf*. wheatlandi, LACM 150177 (https://fishtreeoflife.org/fossils/). The Markov chain was run for 40 million generations, with sampling every 500 generations and a burn‐in of the initial 25% of the samples. The MCMC results were verified using Tracer v.1.7.2, with an Effective Sample Size (ESS) exceeding 200. The phylogenetic trees were visualized using Figtree v1.4.4. Moreover, the whole‐genome average nucleotide identity (ANI) between species was analyzed using FastANI v1.34 (Jain et al. 2018).

### 2.6 High‐Density Haplotype Reference Panel and Characterization of LoF Mutations

Sixty individuals from a candidate base population of our laboratory (CBP‐MGB), which originated from wild individuals (*n* = 450) collected in Guangdong in 2008 and had undergone four generations of selection for growth rate and health status under aquaculture conditions, were used for caudal fin sampling and DNA extraction, followed by sequencing on the DNBSEQ‐T7 platform. The raw sequencing reads were processed through quality filtering using Fastp and subsequently mapped to the reference genome via BWA v0.7.17‐r1188 (Li and Durbin 2010). Duplicated reads were identified and marked using MarkDuplicates of Picard v2.24.0. Variant calling was performed using the HaplotypeCaller and GenotypeGVCFs algorithms within the GATK v4.1.0 (Van der Auwera et al. 2013). Raw Single Nucleotide Polymorphisms (SNPs) were filtered using vcffilter‐rtg v3.12.1 with the parameters “GQ < 20, QD < 10, FS > 10, MQ < 40, ReadPosRankSum < −8, SOR > 4, MQRankSum < −12.5.” Additional filtering excluded SNPs with MAF < 0.01, missing rate > 0.1, and those within 5 bp of other variants using bcftools v1.3.1 (Zeng et al. 2022). Only biallelic SNPs were retained for downstream analyses. The haplotype reference panel was phased with the read‐aware phasing method implemented in SHAPEIT v2.r904 (Delaneau et al. 2013). SnpEff v4.3t was used to identify the loss‐of‐function (LoF; including stop‐gain and splice‐site‐disrupting mutations), missense, and synonymous variations (Cingolani et al. 2014). Functional enrichment for KEGG pathways was conducted using clusterProfiler (Wu et al. 2021), and GO term enrichment was carried out using EnrichPipeline.

## 3 Results

### 3.1 A Complete T2T Genome of *P. leopardus*

High‐molecular‐weight DNA (> 100 kb) was extracted for ultra‐long Oxford Nanopore (UL‐ONT) sequencing. Sequencing using three ONT flow cells generated 161.17 Gb of raw reads, with a read N50 of 23,690 bp and a maximum read length of 1.170 Mb (Table S1). Following length (> 30 kb) and quality (Q20) filtering, 109.85 Gb (~86× coverage) ONT reads were retained, with reads N50 61,809 bp. The filtered dataset underwent read‐level self‐correction using HERRO, preserving 105.56 Gb of high‐fidelity ONT data. PacBio HiFi sequencing yielded 76.27‐Gb reads (> 10 kb), providing ~86× coverage. Hi‐C data generated 209.66 Gb of clean sequences at ~236× coverage.

**FIGURE 1:**
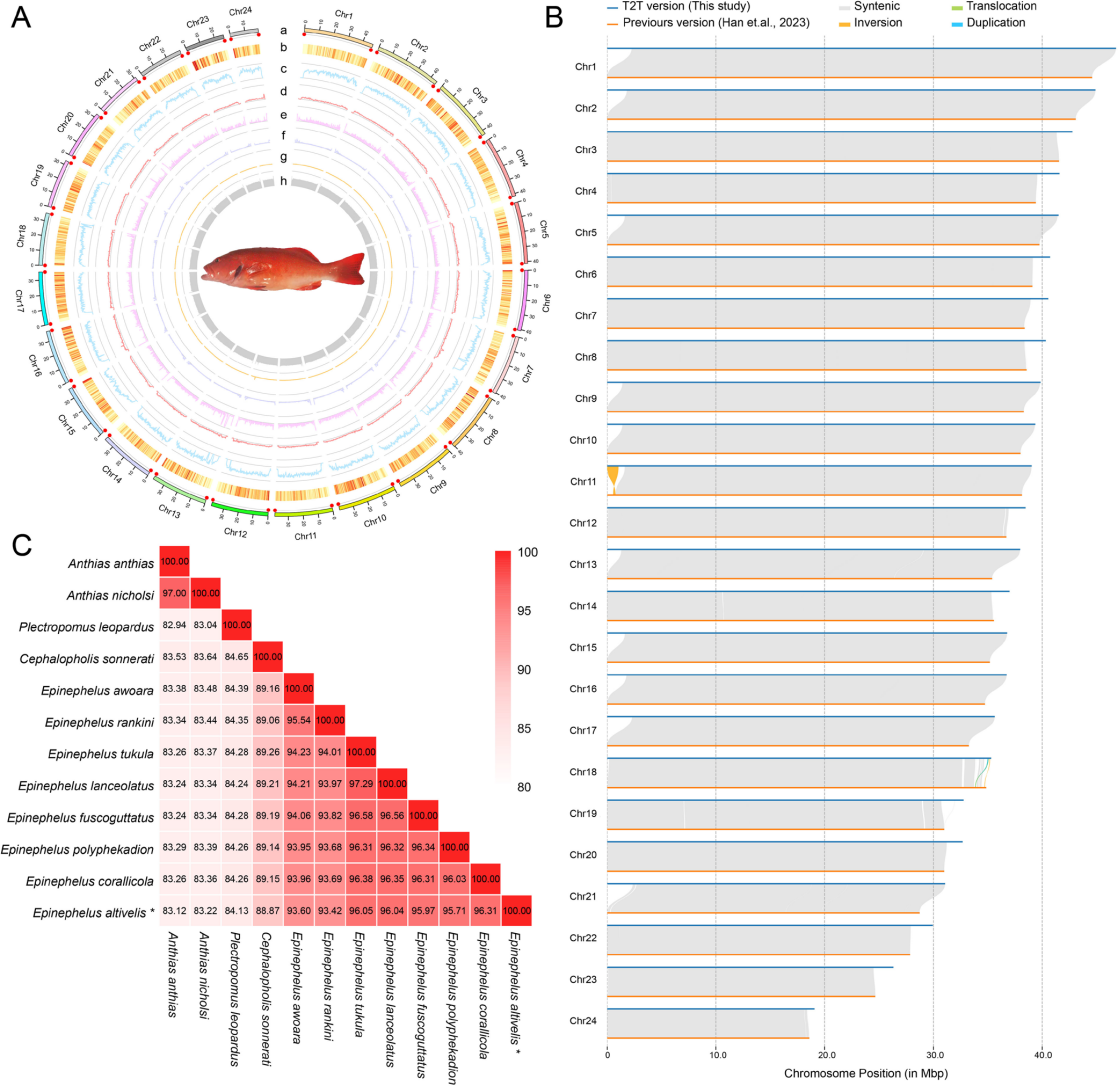
T2T genomes of *P. leopardus* . (A) Chromosomal features of the *P. leopardus* . (a) Telomeres; (b) gene density; (c) SNP density; (d) DNA transposons; (e) LTR; (f) LINE; (g) SINE; (h) GC content. (B) Synteny and structural variation analyses compared to the previous genome version. (C) ANI Analysis among species of Epinephelidae and Anthiadidae. *Denotes the updated taxonomy (*Epinephelus altivelis*, formerly *Cromileptes altivelis* ).

Utilizing these data, six haplotype‐resolved genomes from three assembly versions were generated, each with a contig N50 > 37 Mb (Table S2). To obtain a complete, gapless genome, the best complete chromosome for each chromosome was selected from the six haplotype‐resolved assemblies, resulting in a final genome comprising 24 gapless contigs, each representing a complete chromosome (Figure S1). The final assembly had a genome size of 887.20 Mb with a contig N50 of 39.03 Mb (Table 1). The BUSCO completeness of the genome was 99.4% [S: 99.1%, D: 0.3%] (F: 0.5%, M: 0.1%, E: 2.0%), which was comparable to the level of other high‐quality fish genomes (typically around 99.3%) (Lu et al. 2024; Zhao et al. 2025). The Braker annotation pipeline and annotation lift over ultimately identified 25,413 protein‐coding genes in the *P. leopardus* genome. BUSCO assessment demonstrated high completeness: C: 98.0% [S: 96.8%, D: 1.1%], F: 1.0%, M: 1.0%, consistent with genome evaluation metrics excluding BUSCO genes containing stop codons. Functional annotation revealed 22,544 functionally annotated genes, including 16,388 with GO terms, 16,249 with KEGG pathway annotations, and 21,768 with Pfam domain assignments. The QV of genome assembly achieved 67, whereas the highest QV of chromosome reached 84 (Table S3). Telomeres were identified using the Quartet software, with all 48 telomeres successfully detected, indicating we generated a gapless T2T genome assembly of *P. leopardus* (Figure 1A and Table S3).

**TABLE 1:** Summary and comparison of genome assemblies for *P. leopardus* and related species.

Type	This study^a^	Han et al.	Zhou et al.	*C. sonnerati* (hapA)^a^	*E. corallicola* (hap1)^b^
Assembly statistics					
Genome size (Mb)	887.20	849.74	895.7	1039.95	1086.41
Contig N50 (Mb)	39.03	35.59	0.90	43.83	44.94
Scaffold N50 (Mb)	39.03	38.02	33.28	43.83	45.24
BUSCO completeness (%)	99.4	99.4	94.7	99.3	99.3
Single copy (%)	99.1	99.0	92.3	99	98.8
Duplicated (%)	0.3	0.4	2.4	0.4	0.4
Fragmented (%)	0.5	0.4	2.2	0.6	0.6
Missing (%)	0.1	0.2	3.1	0.1	0.1
Annotation statistics					
BUSCO completeness (%)	98.0	96.0	94.7	94.9	98.0
Single copy (%)	96.8	94.8	92.3	93.2	96.7
Duplicated (%)	1.1	1.3	2.4	1.7	1.4
Fragmented (%)	1.0	1.2	2.2	1.1	0.8
Missing (%)	1.0	2.8	3.1	4.0	1.1

^a^ Represents the complete genome assembly (all chromosomes T2T).

^b^ Represents the near‐complete assembly (≥ 1 chromosome T2T).

Compared to the previously highest‐quality *P. leopardus* genome assembly, the current version showed an increase in genome size by 37.46 Mb and an improvement in contig N50 from 35.59 to 39.03 Mb (Han et al. 2023). Although the BUSCO score for genome completeness did not increase significantly, the BUSCO completeness for protein‐coding gene annotations improved by 2% (Table 1). Synteny and structural variation analyses revealed a high degree of chromosomal collinearity between the two genome versions, demonstrating strong consistency between the assemblies (Figure 1B). Notably, the regions exhibiting assembly improvements were predominantly concentrated at one end of each chromosome.

### 3.2 A Genome‐Wide Phylogenetic Framework for Perciformes

**FIGURE 2:**
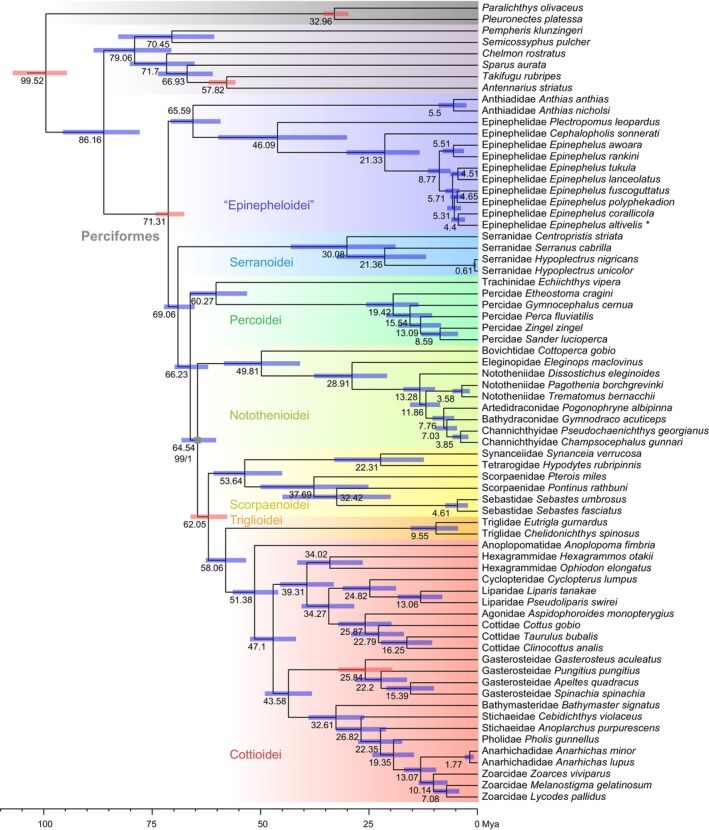
Phylogenetic tree and divergence time estimates of Perciformes. Red bar: calibration node (divergence times based on fossil/molecular clock data). Gray box: node support value of 99% (BS)/1.00 (PP). *Denotes the updated taxonomy (*Epinephelus altivelis*, formerly *Cromileptes altivelis* ).

A total of 31 species were re‐annotated, and the completeness of the re‐annotated genes (BUSCO) ranged from 93.6% to 98.3%, with a median of 96.6% (Supplementary File 2). We identified a total of 1769 single‐copy orthologous genes across the studied 70 species. After trimming non‐conserved regions and filtering out shorter sequences, 1731 single‐copy orthologous genes (comprising 534,763 amino acids) were retained for phylogenetic tree reconstruction. The best‐fit amino acid substitution model, selected under the Bayesian Information Criterion (BIC), was JTT + I + G4 for Maximum Likelihood (ML) analysis, while the WAG + I + G4 model was employed for Bayesian inference in MrBayes. The topology of the maximum likelihood phylogenetic tree was consistent with that of the Bayesian phylogenetic tree. Except for one node with a support value of 99% ML bootstrap (BS) and 1.00 Bayesian posterior probability (PP), all other nodes had support values of 100% (BS) and 1.00 (PP), indicating a highly robust and well‐resolved phylogenetic relationship (Figures 2 and S2).

The internal topology of Cottioidei was composed of six major lineages, forming a clade with the following relationship: ((Zoarcoidea + Gasterosteidae) + ((Cottoidea + Cyclopteroidea) + Hexagrammidae)) + Anoplopomatidae. Triglioidei was recovered as the sister group to Cottioidei, and together they form a clade with Scorpaenoidei. The suborder Notothenioidei was resolved as the outgroup to this clade, with a nodal support value of 99% (BS)/1.00 (PP). Within Notothenioidei, the superfamily Cryonotothenioidea (including Nototheniidae, Channichthyidae, and others) (Near et al. 2015), characterized by the presence of *AFGP* genes, was estimated to have diverged from non‐Antarctic notothenioid species approximately 28.91 Mya. The onset of diversification of the Cryonotothenioidea was estimated at around 13.28 Mya, which aligns well with the Middle Miocene Climate Transition, a period marked by major Antarctic ice sheet expansion and global cooling during the interval 15–13 Mya (Frigola et al. 2018). This estimated diversification timing was slightly earlier than previous estimates (10.70 Mya, 95% confidence interval 14.1–7.8 Mya) (Bista et al. 2023).

Within the Percoidei, phylogenetic topology revealed that Trachinidae and Percidae form a sister group. Serranoidei was reconstructed as non‐monophyletic, with Epinephelidae and Anthiadidae (previously classified as Epinephelinae and Anthiadinae under Serranidae) forming a clade positioned at the base of the Perciformes phylogeny (Rimmer and Glamuzina 2017). This result was consistent with a previous UCE‐based study, though the nodal support was only 51% (BS) in that analysis, whereas this clade received maximum support (100% BS and 1.00 PP) in our phylogeny (Ghezelayagh et al. 2022). The less inclusive delimitation of Serranidae served as an outgroup to other Perciformes excluding Anthiadidae and Epinephelidae. The UCE‐based study reported a clade comprising Percoidei and the less inclusive delimitation of Serranidae, which was not supported by our phylogenetic analyses (Ghezelayagh et al. 2022). Within Epinephelidae, *Plectropomus* was the outgroup to (*Cephalopholis* + *Epinephelus*), with an estimated divergence time of 46.09 Mya. The whole‐genome ANI between *P. leopardus* and the (*Cephalopholis* + *Epinephelus*) clade ranged from 84.13% to 84.65% (Figure 1C). *Cromileptes altivelis* (Valenciennes, 1828), the only species in the genus *Cromileptes*, was nested within Epinephelus, which is consistent with the phylogeny of groupers based on Sanger sequencing (Ma et al. 2016). The whole genome ANI between species of the genus *Cromileptes* and those of the genus *Epinephelus* was higher than the ANI among some species within the genus *Epinephelus* itself.

### 3.3 Evolutionary Dynamics of Repetitive Sequences Across Grouper Genomes

The genome of *P. leopardus* was the smallest among currently available high‐quality grouper genome assemblies, with a size of 887.20 Mb. Compared to *C. sonnerati* (1039.53 Mb) and *E. corallicola* (1086.41 Mb), the differences in genome size were 152.33 and 199.21 Mb, respectively (Figure S3 and Table S4) (Lu et al. 2024; Zhao et al. 2025). Analysis showed that the difference in repetitive sequence length accounted for 115.85 and 138.98 Mb, representing 76.05% and 69.77% of the total genome size differences, respectively. This indicated that variation in repetitive sequence content was the primary factor driving genome size divergence among these species.

**FIGURE 3:**
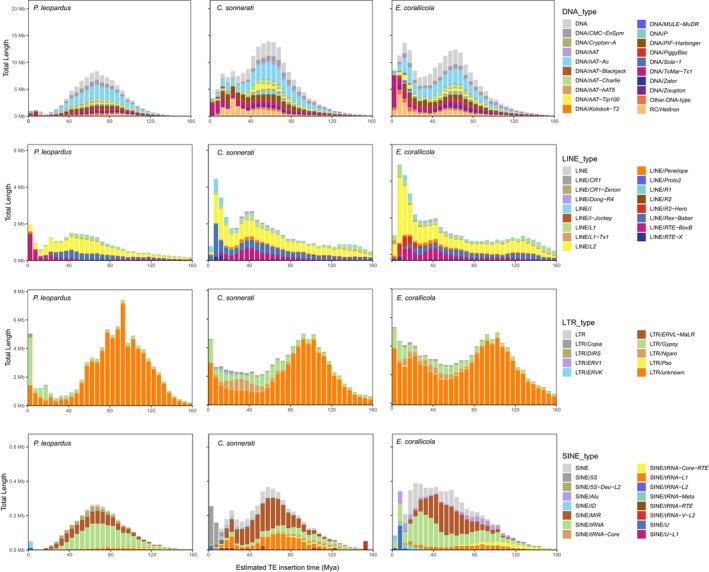
Estimated insertion times of four TE classes among groupers.

Estimates of TE insertion times revealed how transposon activity drove genome size differences and also reflected the divergence history of the species (Figures 3 and S3). Among the four major TE types, *P. leopardus* showed the largest difference in DNA transposon content compared to *C. sonnerati* and *E. corallicola* , mainly due to known DNA types including RC/*Helitron*, DNA/*TcMar‐Tc1*, DNA/*hAT‐Ac*, and DNA/*PIF‐Harbinger* (Table S4 and Supplementary File 3). The observed divergence was attributable to the suppressed activity of DNA transposons in *P. leopardus* from 0 to 45 Mya. In contrast, both *C. sonnerati* and *E. corallicola* exhibited sustained activity of these DNA transposons during this period. Prior to 20 Mya, *C. sonnerati* and *E. corallicola* showed similar levels of DNA transposon activity. After 20 Mya, however, *E. corallicola* underwent a stronger expansion of DNA transposons (DNA/*hAT‐Ac* and DNA/*TcMar‐Tc1*). This timing coincided with the estimated divergence time between *Cephalopholis* and *Epinephelus* (~21.36 Mya), suggesting that the burst may represent an independent expansion of transposons in the Epinephelus lineage after divergence. For LINE, both *Cephalopholis* and *Epinephelus* experienced two major expansion events, at approximately 5–20 and 30–45 Mya. The earlier burst (30–45 Mya), which predated the divergence of the two genera, was primarily driven by LINE/*RTE‐BovB* (Figure 3). During the 5–20‐Mya period, the expansion in *Epinephelus* was mainly attributable to LINE/*L2*, LINE/*RTE‐BovB* and LINE/*R2‐Hero*, whereas in *Cephalopholis* it was dominated by LINE/*Rex‐Babar*, LINE/*L2*, and LINE/*I* (Figure 3). In contrast, *P. leopardus* only exhibited a recent, limited burst of LINE/*RTE‐BovB* during the last 5 million years. Regarding LTR, both *C. sonnerati* and *E. corallicola* showed sustained LTR activity throughout 0–45 Mya, while *P. leopardus* displayed a sharp burst of LTR activity only during the past 5 million years. As for SINE, all species exhibited low SINE sequence content, which contributed minimally to genome size.

### 3.4 High‐Density Haplotype Reference Panel and Characterization of LoF Mutations

**FIGURE 4:**
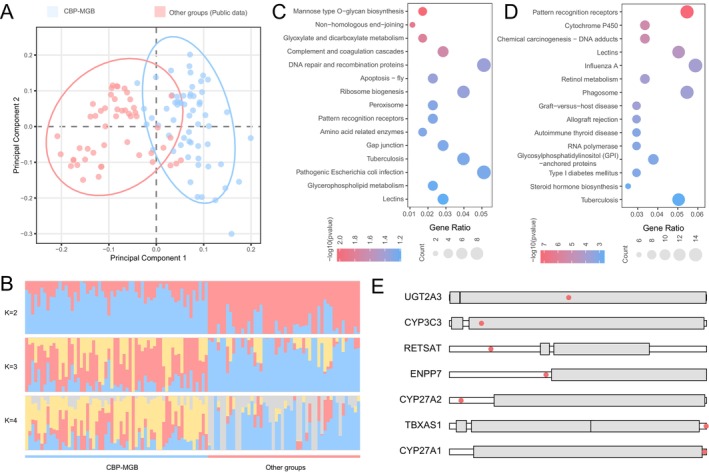
Analysis of haplotype reference panel of *P. leopardus* . (A) PCA analysis of 110 individuals. (B) Genetic structure analysis. (C) KEGG enrichment of splice‐site‐disrupting Lof mutations. (D) KEGG enrichment of stop‐gain Lof mutations. (E) Localizations of stop‐gain mutations within the protein‐coding regions of genes involved in Retinol metabolism pathway. Grey box: denotes the domain of protein‐coding sequences. Red dot: represents the relative position of stop‐gain Lof mutations in protein‐coding sequences.

A total of 60 individuals from a candidate base population (CBP‐MGB) were subjected to whole‐genome resequencing, which yielded sequence data volumes ranging from 16.62 to 22.2 Gb, with an average depth of approximately 20×. These data were integrated with previously published sequencing data from 50 individuals (public dataset, 9.84–21.08 Gb, average depth ~14×) to construct a combined reference panel (Zhou et al. 2020). After quality control, a final panel of 10,596,146 high‐quality SNPs was obtained, with an average density of one SNP per 83.73 bp. Principal component analysis (PCA) and genetic structure analysis revealed that the CBP‐MGB and the public dataset formed genetically distinct sub‐populations (Figure 4A,B).

Comprehensive functional annotation of the haplotype reference panel was performed to determine the location and predicted biological impact of each SNP. This analysis identified 217,684 synonymous and 108,662 nonsynonymous SNPs. Additionally, we detected a total of 1068 LoF mutations, comprising 702 stop‐gain mutations and 366 splice‐site‐disrupting mutations (Table 2). These LoF events affected 652 and 352 protein‐coding genes, respectively. Functional enrichment indicated that genes with splice‐site‐disrupting mutations were significantly enriched in the KEGG pathways of mannose‐type O‐glycan biosynthesis, non‐homologous end‐joining and glyoxylate and dicarboxylate metabolism (Figure 4C), as well as the GO terms for striated muscle myosin thick filament (GO: 0005863) and kinin cascade (GO: 0002254) (Supplementary File 4). The genes with stop‐gain mutations were significantly enriched in immune‐related functions, including the pattern recognition receptors KEGG pathway (Figure 4D), as well as the GO terms: defense response to other organism (GO: 0098542) and regulation of immune response (GO: 0050776) (Supplementary File 5). These genes mainly included *MR1*, *MHCII‐DPB1*, *TRIM7*, *TRIM35*, *NLRP1*, *NLRP12*, *TLR2*, and *TLR22*. Notably, we observed a significant enrichment of stop‐codon gain mutations in the Retinol metabolism pathway, which may be associated with skin color variation (Yang et al. 2020). In this pathway, the stop‐gain mutations in *UGT2A3* and *CYP3C3* occurred within functional domains, suggesting a fundamental disruption of protein function (Figure 4E). Mutations in *RETSAT*, *ENPP7*, and *CYP27A2* were located upstream of any functional domain regions, whereas those in *TBXAS1* and *CYP27A1* were found near the native stop‐codon.

**TABLE 2:** Annotation of SNP reference panel of *P. leopardus* .

Category	Type	Count
Region	DOWNSTREAM	1,723,619
	EXON	322,679
	INTERGENIC	4,518,910
	INTRON	5,440,770
	SPLICE_SITE_ACCEPTOR	162
	SPLICE_SITE_DONOR	204
	SPLICE_SITE_REGION	36,565
	UPSTREAM	1769,768
	UTR_3_PRIME	267,846
	UTR_5_PRIME	56,616
Type	missense_variant	108,662
	start_lost	143
	stop_gained	702
	stop_lost	127
	synonymous_variant	217,684
Total	—	10,596,146

## 4 Discussion

With the rapid advancement of sequencing technologies and the application of deep learning in sequencing (Hall et al. 2024), the read length and accuracy of ONT have continued to improve, greatly facilitating the assembly of T2T genomes. In this study, we combined highly accurate ONT and PacBio HiFi reads using the Raft‐HiFiasm and standard Hifiasm pipelines to generate a complete T2T genome for *P. leopardus* without the need for gap‐filling. Given its high completeness and low base error rate, this assembly qualifies as the representative reference genome for this species. Compared to the previously published genome assembly, the total genome size increased by 37.46 Mb. However, the genome BUSCO assessment did not show significant improvement, indicating that the increased regions primarily consisted of non‐coding sequences. Intriguingly, comparative analysis with the previous genome assembly revealed that the increased regions were consistently located at one end (either left or right) of each chromosome, and this pattern was observed across all chromosomes. These regions were enriched with LTR and LINE. We hypothesized that this phenomenon was not coincidental but rather represented the structural characteristics (complex regions unresolved by previous assembly) of the chromosomes in this species, as uncovered by the UL‐ONT‐assembled T2T genome. In addition, the number of high‐quality genomes of groupers has been gradually increasing, and the genome BUSCO scores appear to stabilize at around 99.3%–99.4% (Lu et al. 2024; Zhao et al. 2025), which may represent the upper limit of grouper genome BUSCO (actinopterygii_odb10 v2024‐01‐08).

Previously, the systematics of the Perciformes underwent significant revision, and the composition of its major suborders is widely accepted (Betancur‐R et al. 2017). However, phylogenetic relationships among these suborders and within various families often show inconsistencies across studies, particularly for Serranoidei, which includes Epinephelidae (Betancur‐R et al. 2017; Ghezelayagh et al. 2022; Hughes et al. 2018). In our study, we recovered a topology structured as (Epinephelidae + Anthiadidae) + (Serranoidei + (Percoidei + (Notothenioidei + (Triglioidei + (Scorpaenoidei + Cottioidei))))). Epinephelidae and Anthiadidae form a monophyletic clade situated at the base of the Perciformes, indicating that Serranoidei, as traditionally defined, is non‐monophyletic. Although recent studies based on UCE data have separated Epinephelidae and Anthiadidae from Serranoidei, this clade has not been well resolved (Ghezelayagh et al. 2022). Our phylogenetic tree, based on the whole‐genome matrix, reliably resolved the position of this clade within Perciformes. We suggest recognizing this basal clade, comprising Epinephelidae and Anthiadidae, as a distinct suborder separate from Serranoidei, and designating it as “Epinepheloidei” (WoRMS id: 1860372). This revision will help to reduce confusion surrounding subordinal classifications within Perciformes and clarify the phylogenetic status of the economically important Epinephelidae (Rimmer and Glamuzina 2017). Nevertheless, delineating the exact boundaries of this new suborder requires expanded taxonomic sampling in future phylogenetic studies, particularly for the genus *Acanthistius* (Ghezelayagh et al. 2022). Within the family Epinephelidae, *C. altivelis* is the only species within the genus *Cromileptes*. Phylogenetically, this species is nested within the genus *Epinephelus* and forms a sister clade to *E. corallicola* . The recognition of *Cromileptes* as a distinct genus was not supported by phylogenetic or ANI analyses. Maintaining *Cromileptes* as a separate genus might be inappropriate and could lead to misconceptions in grouper hybridization breeding programs (Cao et al. 2024). Based on phylogenetic evidence, we propose that *Cromileptes* should be synonymized under *Epinephelus*, with *Cromileptes altivelis* reclassified as *Epinephelus altivelis* (Valenciennes, 1828). Additionally, although genome data are currently available for only a limited number of species, our findings still revealed non‐monophyletic relationships in several families, such as Stichaeidae, Scorpaenidae, and Nototheniidae. This indicates that broader sampling and revisions remain necessary for phylogenetic studies at the family level within the Perciformes.

The construction of a haplotype reference panel is crucial for species conservation and breeding programs (Ciezarek et al. 2022; Zeng et al. 2022). In this study, we constructed a haplotype reference panel based on the complete T2T genome using 110 individuals. Our analysis of genetic variants specifically focused on LoF mutations. Genes with stop‐gain mutations were primarily involved in immune‐related functions. LoF mutations in these genes may contribute to inter‐individual differences in disease resistance (Quintana‐Murci et al. 2014). Therefore, monitoring these loci may hold significant relevance for research on disease resistance in *P. leopardus* . For instance, incorporating these LoF mutation sites into the SNP panel for genomic selection of disease resistance could potentially improve prediction accuracy (Li et al. 2024). Additionally, we observed significant enrichment of stop‐gain LoF mutations in the retinol metabolism pathway. In *P. leopardus* , market price is correlated with skin color, making the vibrant red coloration a key target trait in selective breeding programs (Shimose and Kanaiwa 2022). Fish skin coloration is produced by pigment granules in chromatophores and structural color generated by light‐reflecting iridophores and leucophores (Pegu and Singh 2025). Red coloration is produced by carotenoids and pteridines, and cells that contain excessive amounts of red/orange carotenoids are called erythrophores. Therefore, LoF mutations in genes involved in the retinol metabolism pathway are predicted to affect this process and potentially contribute to variations in skin coloration (Xu et al. 2023; Yang et al. 2020). Not all stop‐gain LoF mutations lead to complete functional knockout. For instance, mutations in *TBXAS1* and *CYP27A1* occurred only near the stop codon and did not disrupt the major structural domains of the proteins. The extent to which such mutations affect protein function remains unclear. LoF mutations represent a component of genetic diversity, playing a crucial role in environmental adaptation and species diversification (Xu and Guo 2020), while also serving as valuable resources for genetic breeding. Elucidating these significant genetic variants will not only provide important insights for the selective breeding of *P. leopardus* but also hold important implications for the conservation of species diversity.

## 5 Conclusion

In this study, we present a complete T2T genome assembly for *Plectropomus leopardus* , an economically important species. This high‐quality genome (887.20 Mb; contig N50: 39.03 Mb; QV: 67) qualifies as the definitive reference genome for this species. Furthermore, by integrating and performing large‐scale re‐annotation of publicly available genomic resources, we reconstructed a robust genome‐level phylogenetic framework for the order Perciformes. We also constructed a high‐density haplotype reference panel based on 110 individuals and annotated 1068 loss‐of‐function (LoF) mutations. The genomic resources provided in this study will not only facilitate conservation and genetic breeding programs for *P. leopardus* but also offer fundamental insights into the phylogeny of Perciformes.

## Funding

This work was supported by the National Key Research and Development Program of China (2022YFD2400501), the Fundamental Research Funds for the Central Universities (202562017), the National Natural Science Foundation of China (U24A20457), the Key R&D Project of Hainan Province (ZDYF2024XDNY275 and ZDYF2024XDNY248) and the China Postdoctoral Science Foundation (2024M753060).

## Ethics Statement

This study was approved by the Institutional Animal Care and Use Committee of the College of Marine Life Sciences, Ocean University of China (Project Identification Code: 20240810A2).

## Conflicts of Interest

The authors declare no conflicts of interest.

## Supporting information


**Figure S1:** Hi‐C interaction heatmap of 24 chromosomes of T2T version of *P. leopardus* .

**Figure S2:** Phylogenetic tree of Perciformes. Gray box: node support value of 99% (BS)/1.00 (PP). *Denotes the updated taxonomy (*Epinephelus altivelis*, formerly *Cromileptes altivelis* ).

**Figure S3:** Evolution of repetitive sequences. (A) Composition of repetitive sequences of groupers. (B) Comparison of estimated insertion times of four TE classes among groupers.

**Table S1:** Statistics of long‐read and Hi‐C sequencing data.
**Table S2:** Statistics of genome assemblies of different versions.
**Table S3:** QV and telomere statistics of the T2T genome of P. leopardus.
**Table S4:** Composition of repetitive sequences in grouper genomes.

**Supplementary File 1.** Genome assembly commands.

**Supplementary File 2.** Genomic data resources and BUSCO‐based annotation statistics for phylogenomics analysis.

**Supplementary File 3.** Composition of TE subtype in grouper genomes.

**Supplementary File 4.** GO enrichment analysis of genes with splice‐site‐disrupting mutations.

**Supplementary File 5.** GO enrichment analysis of with stop‐gain mutations.

## Data Availability

The raw sequencing data and genome assemblies for *P. leopardus* have been deposited at the National Center for Biotechnology Information (NCBI) under BioProject accession PRJNA1283370. Additionally, the annotation files and haplotype reference panel for *P. leopardus* , along with re‐annotation files (31 species) for Perciformes, are available on FigShare (https://figshare.com/s/ab615dbd4e5237d914e4 and https://figshare.com/s/eab13f9aaeaa48f4ee4e).
