# Supplementary figures and images for: A Complete Telomere‐To‐Telomere Assembly of Plectropomus leopardus and Phylogenomic Insights Into Perciformes
Source: Evol Appl. 2026 Jul 9;19(7):e70296. doi: 10.1111/eva.70296 (PMC13351114; doi:10.1111/eva.70296)

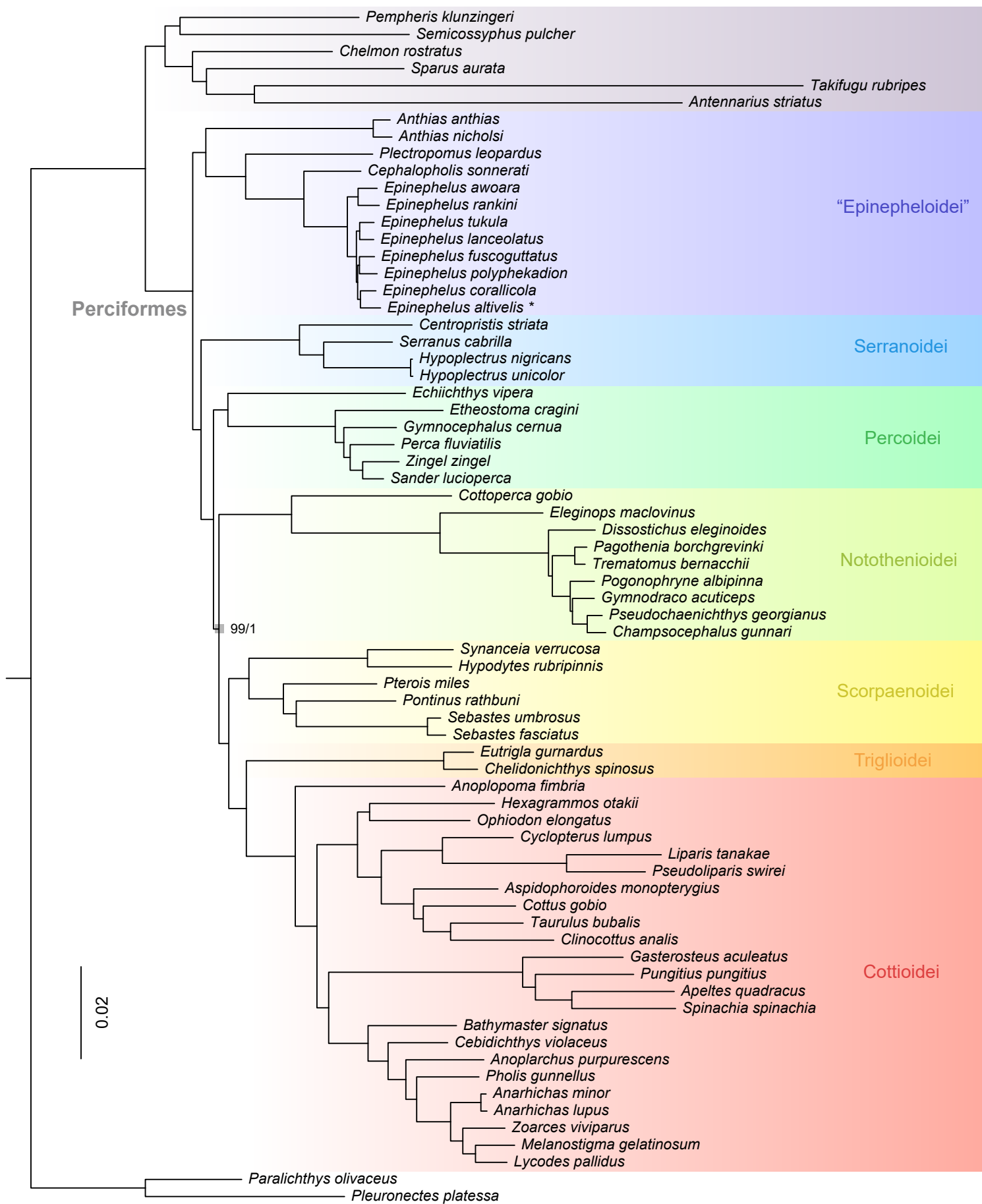

Supplement: Supplementary file 2 — Figure S2: Phylogenetic tree of Perciformes. Gray box: node support value of 99% (BS)/1.00 (PP). *Denotes the updated taxonomy (Epinephelus altivelis, formerly Cromileptes altivelis ). [file EVA-19-e70296-s005.pdf]

A

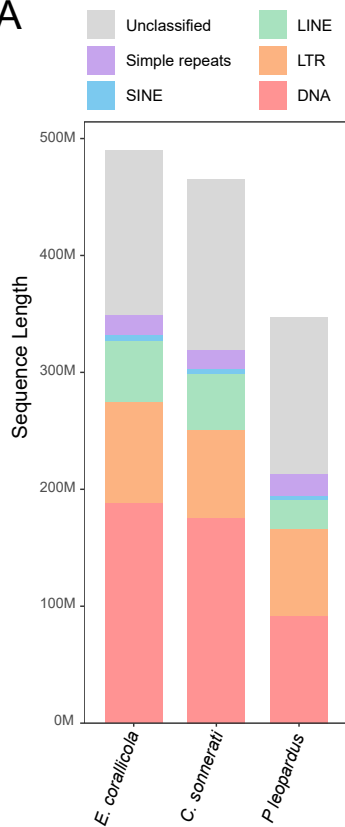

B

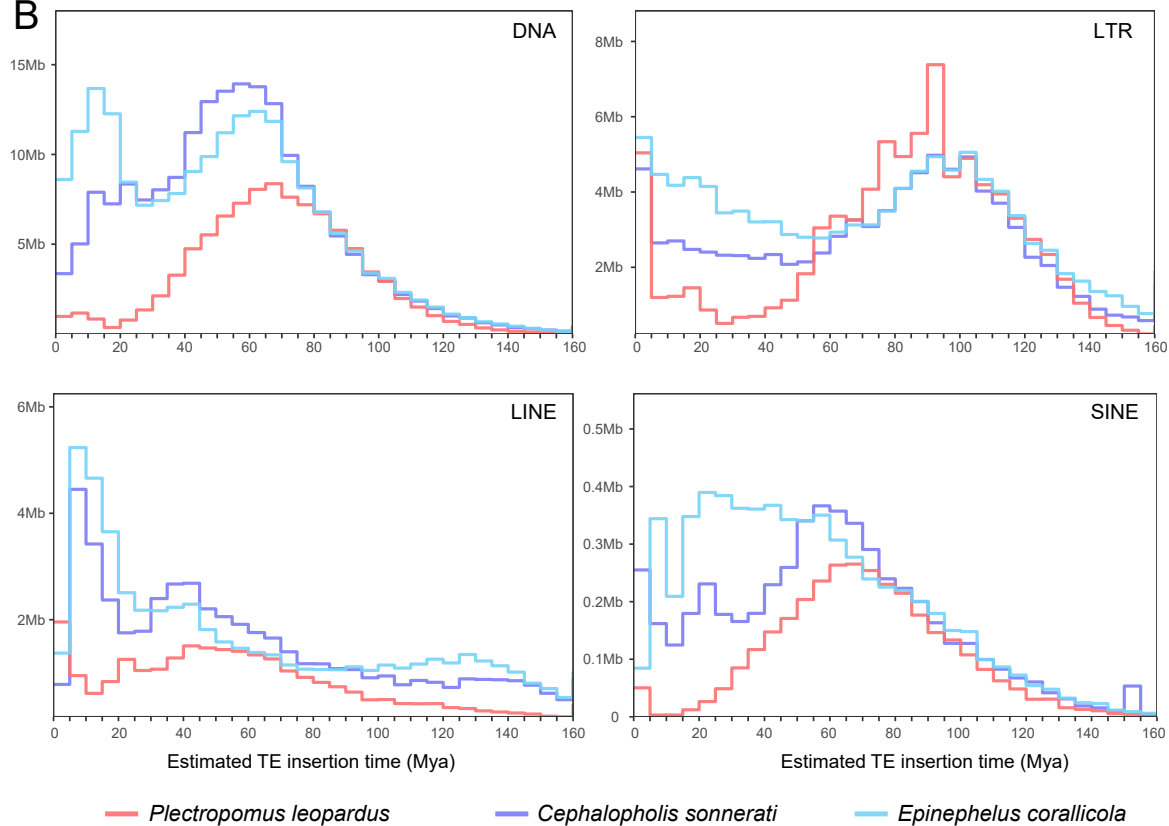

Supplement: Supplementary file 3 — Figure S3: Evolution of repetitive sequences. (A) Composition of repetitive sequences of groupers. (B) Comparison of estimated insertion times of four TE classes among groupers. [file EVA-19-e70296-s009.pdf]
