# Supplementary material for: A Complete Telomere‐To‐Telomere Assembly of Plectropomus leopardus and Phylogenomic Insights Into Perciformes
Source: Evol Appl. 2026 Jul 9;19(7):e70296. doi: 10.1111/eva.70296 (PMC13351114; doi:10.1111/eva.70296)
Supplement: Supplementary file 4 — Table S1: Statistics of long‐read and Hi‐C sequencing data. Table S2: Statistics of genome assemblies of different versions. Table S3: QV and telomere statistics of the T2T genome of P. leopardus. Table S4: Composition of repetitive sequences in grouper genomes. [file EVA-19-e70296-s008.docx]

Table S1 Statistics of long-read and Hi-C sequencing data.

| Reads Type | Data size (bp) |
| --- | --- |
| Pacbio HiFi Reads (>10kb) | 76,272,033,187 |
| Pacbio HiFi Reads (>13kb) | 55,436,202,240 |
| ONT Row Reads | 161,168,467,430 |
| ONT (>30kb Q20) | 109,849,022,944 |
| HERRO Corrected Reads | 105,559,433,355 |
| HiC Reads | 209,656,258,200 |

Table S2 Statistics of genome assemblies of different versions

| Version | Contig N50 (Mb) | Genome Size  (Mb) | Chromosomes included in the final Assembly |
| --- | --- | --- | --- |
| V1_hap1 | 39.03 | 887.17 | 13, 18, 22 |
| V1_hap2 | 37.72 | 897.72 | 2,3,5,7,9,10,15,17, 20,21,24 |
| V2_hap1 | 39.19 | 897.56 | 14, 23 |
| V2_hap2 | 39.03 | 881.79 | 11,16 |
| V3_hap1 | 37.96 | 886.14 | 1,4 |
| V3_hap2 | 39.20 | 894.40 | 6,8,12,19 |

| Chromosome | Length (bp) | Error bases | QV | Left  telomere | Right telomere |
| --- | --- | --- | --- | --- | --- |
| Chr1 | 46773224 | 328 | 64.7634 | 668 | 481 |
| Chr2 | 44892086 | 278 | 65.3034 | 1284 | 780 |
| Chr3 | 42786272 | 49 | 72.6333 | 1205 | 486 |
| Chr4 | 41590218 | 231 | 65.776 | 810 | 414 |
| Chr5 | 41507094 | 97 | 69.5357 | 727 | 1737 |
| Chr6 | 40716968 | 54 | 71.996 | 527 | 530 |
| Chr7 | 40554911 | 134 | 68.0316 | 824 | 641 |
| Chr8 | 40331527 | 26 | 75.1289 | 360 | 1217 |
| Chr9 | 39854020 | 350 | 63.7862 | 1105 | 786 |
| Chr10 | 39357923 | 149 | 67.4406 | 1002 | 1211 |
| Chr11 | 39025492 | 3 | 84.3645 | 1205 | 751 |
| Chr12 | 38476440 | 38 | 73.2763 | 508 | 656 |
| Chr13 | 37987955 | 24 | 75.2165 | 976 | 1471 |
| Chr14 | 36999448 | 37 | 73.2221 | 817 | 486 |
| Chr15 | 36782338 | 42 | 72.6461 | 711 | 691 |
| Chr16 | 36739695 | 43 | 72.5389 | 1214 | 1226 |
| Chr17 | 35640224 | 182 | 66.1409 | 1266 | 389 |
| Chr18 | 35318009 | 118 | 67.9833 | 728 | 119 |
| Chr19 | 32773583 | 152 | 66.559 | 259 | 942 |
| Chr20 | 32686431 | 51 | 71.2902 | 1391 | 1029 |
| Chr21 | 31073787 | 29 | 73.5222 | 1257 | 626 |
| Chr22 | 29932794 | 51 | 70.908 | 1002 | 751 |
| Chr23 | 26328594 | 593 | 59.6959 | 730 | 865 |
| Chr24 | 19073468 | 411 | 59.888 | 1141 | 1037 |

Table S3 QV and telomere statistics of the T2T genome of *P. leopardus*

| Category | *P. leopardus^1^* | *C. sonnerati* (hapA) | *E. corallicola* (hap1) |
| --- | --- | --- | --- |
| SINEs (Mb) | 3.08 | 4.92 | 5.91 |
| LINEs (Mb) | 25.43 | 47.73 | 51.16 |
| LTRs (Mb) | 73.78 | 74.44 | 86.99 |
| DNA transposons (Mb) | 91.67 | 175.52 | 184.26 |
| Unknown TEs (Mb) | 134.30 | 146.86 | 140.86 |
| Small RNA (Mb) | 1.78 | 0.99 | 2.80 |
| Satellites (Mb) | 3.82 | 0.83 | 2.86 |
| Simple repeats (Mb) | 18.38 | 15.76 | 16.82 |
| Low complexity (Mb) | 2.51 | 2.46 | 2.38 |
| Total Repeat sequences (Mb) | 353.39 | 469.24 | 492.37 |
| Genome Size (Mb) | 887.20 | 1,039.53 | 1086.40 |

Table S4 Composition of repetitive sequences in grouper genomes
