# Supplementary material for: A Complete Telomere‐To‐Telomere Assembly of Plectropomus leopardus and Phylogenomic Insights Into Perciformes
Source: Evol Appl. 2026 Jul 9;19(7):e70296. doi: 10.1111/eva.70296 (PMC13351114; doi:10.1111/eva.70296)
Supplement: Supplementary file 5 — Supplementary File 1. Genome assembly commands. [file EVA-19-e70296-s006.docx]

**Supplementary_file 1: Genome assembly commands**

dorado basecaller $model --trim all --emit-fastq Row.pod5 > Row.fastq

seqkit seq -Q $base_quality -M $length Row.fastq > Filter.fastq

dorado correct -m $correct_model Filter.fastq -t $threads > Herro-ont.fasta

Raft_pipline

hifiasm -o errorcorrect -t128 --write-ec ${ont} --h1 ${h1} --h2 ${h2} 2> errorcorrect.log

COVERAGE=$(grep "homozygous" errorcorrect.log | tail -1 | awk '{print $6}')

echo "$COVERAGE" > coverage

hifiasm -o getOverlaps -t128 --dbg-ovec errorcorrect.ec.fa 2> getOverlaps.log

cat getOverlaps.0.ovlp.paf getOverlaps.1.ovlp.paf > overlaps.paf

raft -l ${length} -e ${COVERAGE} -o fragmented errorcorrect.ec.fa overlaps.paf

seqkit fq2fa Name.fastq > Name.fa

hifiasm -o v1 -t 110 HiFi(>13kb)+Raft-Herro-ont.fa --h1 Hic_R1.fastq.gz --h2 Hic_R2.fastq.gz --ul Herro_ONT.fa --telo-m CCCTAA

hifiasm -o v2 -t 110 HiFi(>15k)+Raft-Herro-ont(20x).fa --h1 Hic_R1.fastq.gz --h2 Hic_R2.fastq.gz --ul Herro_ONT.fa --telo-m CCCTAA -s 0.50 -D 6 -N 105

hifiasm -o v3 -t 110 HiFi(10kb).fastq --h1 Hic_R1.fastq.gz --h2 Hic_R2.fastq.gz --ul ONT(>Q20).fastq --telo-m CCCTAA -D 6 -N 105 -s 0.50
